# γ-Glutamyl-Transpeptidase-Resistant Glutathione Analog Attenuates Progression of Alzheimer’s Disease-like Pathology and Neurodegeneration in a Mouse Model

**DOI:** 10.3390/antiox10111796

**Published:** 2021-11-10

**Authors:** Ye In Christopher Kwon, Wei Xie, Haizhou Zhu, Jiashu Xie, Keaton Shinn, Nicholas Juckel, Robert Vince, Swati S. More, Michael K. Lee

**Affiliations:** 1 Department of Neuroscience, University of Minnesota, Minneapolis, MN 55455, USA; YIKWON6@mgh.harvard.edu (Y.I.C.K.); shinn076@umn.edu (K.S.); jucke009@umn.edu (N.J.); 2 Center for Drug Design, College of Pharmacy, University of Minnesota, Minneapolis, MN 55455, USA; wxie@umn.edu (W.X.); zhuhz@mail.uc.edu (H.Z.); jxie@umn.edu (J.X.); vince001@umn.edu (R.V.); 3 Institute for Translational Neuroscience, University of Minnesota, Minneapolis, MN 55455, USA

**Keywords:** glyoxalase 1, oxidative stress, Alzheimer’s disease, progressive neurodegeneration, neuroinflammation, advanced glycation end products (AGEs)

## Abstract

Oxidative stress in Alzheimer’s disease (AD) is mediated, in part, by the loss of glutathione (GSH). Previous studies show that γ-glutamyl transpeptidase (GGT)-resistant GSH analog, Ψ-GSH, improves brain GSH levels, reduces oxidative stress markers in brains of APP/PS1 transgenic mice, a mouse model of AD, and attenuates early memory deficits in the APP/PS1 model. Herein, we examined whether Ψ-GSH can attenuate the disease progression when administered following the onset of AD-like pathology in vivo. Cohorts of APP/PS1 mice were administered Ψ-GSH for 2 months starting at 8 month or 12 months of age. We show that Ψ-GSH treatment reduces indices of oxidative stress in older mice by restoration of enzyme glyoxalase-1 (Glo-1) activity and reduces levels of insoluble Aβ. Quantitative neuropathological analyses show that Ψ-GSH treatment significantly reduces Aβ deposition and brain inflammation in APP/PS1 mice compared to vehicle-treated mice. More importantly, Ψ-GSH treatment attenuated the progressive loss of cortical TH+ afferents and the loss of TH+ neurons in the locus coeruleus (LC). Collectively, the results show that Ψ-GSH exhibits significant antioxidant activity in aged APP/PS1 mice and chronic Ψ-GSH treatment administered after the onset of AD pathology can reverse/slow further progression of AD-like pathology and neurodegeneration in vivo.

## 1. Introduction

Alzheimer’s disease (AD) is the most common neurodegenerative disease, afflicting millions of individuals and carrying a significant economic burden worldwide [1]. AD is characterized by dementia and degeneration of multiple neuronal populations where β-amyloid (Aβ)-related abnormalities are early and necessary pathogenic events [2]. Despite significant effort, currently approved treatments for AD are symptomatic and do not impede the progression of the disease [3]. Thus, there is an urgent need to find new therapies for AD, and the development of successful therapeutics that could delay or modify AD.

Oxidative stress has long been implicated in AD pathogenesis. It is well established that signs of oxidative stress, such as increased accumulation of oxidized cellular components and reduced antioxidant levels, are present in the AD-afflicted brain [4]. A potentially pathogenic oxidized cellular component is the oxidized sugars. Oxidized sugars (OxSu), also termed “reactive dicarbonyls”, for example methylglyoxal (MG), react with proteins, including Aβ and tau, and form proteolysis-resistant advanced glycation end products (AGEs) [5,6]. Such AGEs accelerate the aggregation of Aβ and tau [7,8], eventually triggering neuronal inflammation; hallmarks of Alzheimer’s disease (AD) [9]. The importance of glycation-induced pathogenic processes in AD is also consistent with the increasing link between diabetes and AD, where AD is often considered the Type 3 diabetes [10]. MG is removed by the glyoxalase (Glo) enzyme system of the body that employs glutathione (GSH) as a cofactor, and thus prevents the formation of AGEs. Significantly, decrease in brain Glo activity is seen in human AD [11] and depletion of Glo activity has been shown to facilitate cytotoxicity, increase levels of AGEs, and promote appearance of AD pathologies [12]. Although Glo expression is reduced at advanced stages of AD, reduced Glo activity in brains of early AD cases and AD models is not due to loss of Glo protein. In fact, increased glyoxalase-1 (Glo-1) mRNA and protein are detected in early AD subjects [11]. Thus, at early AD stages, the depletion of Glo-1 activity is likely due to the progressive decrease in the levels of cofactor GSH during aging and AD [11]. Thus, therapeutic supplementation of GSH to increase GSH levels in the brain would reduce oxidative stress and improve Glo function [13]. However, poor bioavailability of GSH due to its cleavage by the omnipresent γ-glutamyl transpeptidase (GGT) has limited the effectiveness of GSH supplementation [14]. Similarly, attempts to promote GSH synthesis by providing precursors also failed due to inadequate biosynthetic machinery in the aging brain [15]. We formulated and successfully tested a solution to this problem through the design and synthesis of a metabolically stable GSH analog, ψ-GSH (Figure S1), which is (a) resistant to cleavage by GGT, (b) able to utilize the GSH influx transporters at the Blood Brain Barrier (BBB), (c) substitute for GSH as a cofactor in Glo (and other GSH dependent) enzyme reactions, and (d) act as an antioxidant against H_2_O_2_ insult [16].

Our lead GGT-resistant GSH-analogue, ψ-GSH, protects cells from Aβ toxicity and reduces AD-related pathology (Aβ deposition, cognitive deficits, and oxidative stress) in the APP/PS1 mouse model of AD [16,17]. In the prior study, the ψ-GSH treatment was initiated in the 3 mo-old APP/PS1 model, well prior to the onset of neuropathology, and the animals were analyzed at a very early stage of the disease (6 months of age). Thus, while this prior study indicated that ψ-GSH can delay the onset of amyloid pathology and memory deficit, it is unknown if ψ-GSH can attenuate the later stages of AD pathology, including inflammation and progressive neurodegeneration, in vivo. Such results will have translational importance as the therapeutic application in humans will likely involve the ψ-GSH treatment being initiated after the onset of significant AD-like pathology. In this report, we now show that ψ-GSH treatment can indeed attenuate signs of oxidative stress, AD-like pathology, and neurodegeneration in aged APP/PS1 mice with established AD-like pathology. Our results suggest that supplementation with ψ-GSH represents a promising therapeutic approach for delaying both the onset and progression of AD.

## 2. Materials and Methods

### 2.1. Mice

We used APPswe/PS1ΔE9 (APP/PS1) mice where both transgenes co-segregate [18] and are congenic on C57Bl6 background (B6.Cg-Tg(APPswe, PSEN1dE9)85Dbo/Mmjax). These mice show progressive cognitive deficits following the onset of amyloid pathology [19] and progressive degeneration of MAergic neurons [20]. Non-transgenic (NTG) littermates were used as controls. All animals were kept on a 12 h-12 h light-dark cycle, with a regular feeding and cage-cleaning schedule. Mice were randomly selected to study groups based on their age and genotype.

### 2.2. ψ-GSH Treatment in Symptomatic APP/PS1 Mice

A cohort of 8 months-old mice was treated either with ψ-GSH (*n* = 4; 500 mg/kg in sterile saline) or saline (*n* = 5) by intraperitoneal (ip) injection. Another independent cohort of 12 months-old APP/PS1 mice was treated either with ψ-GSH (*n* = 8) or saline (*n* = 7) by ip injection. Mice were treated 3 times a week for 8 weeks, at which point they were sacrificed. Equal number of males and females were used as much as possible.

### 2.3. Immunohistochemistry

We followed a protocol described previously [20,21]. Briefly, for histopathological analyses, mice were sacrificed and perfused intracardially with 4% paraformaldehyde and cryoprotected in 30% sucrose solution. Frozen coronal sections (40 μm) were cut using a freezing sliding microtome (Leica, Wetzlar, Germany) and serially distributed into individual wells of 12-well plates. To detect antigens of interest, the sections were incubated in primary antibodies followed by the ABC method (Vector Laboratories, Burlingame, CA, USA) using the chromogen, 3,3′-diaminobenzidine (DAB; Sigma Aldrich, St. Louis, MO, USA) for visualization. Antigen retrieval was performed using a Rodent DeCloaker (BioLegend, San Diego, CA, USA) for all samples, with an additional 88% formic acid pre-treatment for detecting Aβ plaque markers. Primary antibodies used are: clone 4G8 anti-Aβ_17–24_ mouse monoclonal antibody (BioLegend), clone 12F4 anti-Aβ_x−42_ mouse monoclonal antibody (BioLegend), GFAP anti-rabbit polyclonal antibody (Dako, Glostrup Kommune, Denmark), Iba1 anti-rabbit polyclonal antibody (Wako, Japan), and TH (tyrosine hydroxylase) anti-rabbit polyclonal antibody (Millipore, Burlington, MA, USA). Diluted cresyl violet (CV) was used to counterstain nuclei of neurons.

### 2.4. Stereological Analyses of Aβ Pathology and Glial Activation

Stereological analyses were done using the Stereo Investigator software (Micro Bright Field; Colchester, VT) [20,21]. The extent of brain area covered by Aβ deposits (4G8 and 12F4), reactive astrocytes (GFAP), and microglia (Iba1) within the regions of interest (ROI) was measured using the area fraction fractionator probe [22]. The ROIs were determined using The Mouse Brain Stereotaxic Coordinates [23] as the reference. These include the barrel field region of primary somatosensory cortex (S1BF; sections between bregma −0.10 to −1.22 mm, posterior to the anterior commissure and anterior to hippocampus) and dorsal hippocampus (dentate, CA1, CA2/3; sections between bregma −1.46 to −2.18 mm); the S1 barrel cortex (S1BF) and dorsal hippocampal regions. For each animal, we immunostained every 12th coronal brain section (4–6 sections) containing the ROIs and analyzed them using Stereo Investigator (MBF Bioscience, Williston, VT, USA), with a ×40 objective. Within the outlined ROIs, the area fraction fractionator probes was used to systematically and randomly allocate sampling sites 400 μm apart in the cortex, and 200 μm apart in the hippocampus. Counting frame of 100 × 80 μm, containing markers equally spaced from one another at a distance of 15 μm, was used. The area fraction markers that overlap with immunoreactive areas were labeled as positive, whereas remaining markers were labeled negative. Because the ratio of untainted and stained markers is proportional to the areas occupied, we calculated the percent of total area that was immunostained. Representative images are shown in the paper. The images of all brain sections used for the quantitative analysis are available upon request directed to Dr. M.K. Lee.

### 2.5. Quantitative Analyses of Noradrenergic (NAergic) Afferents and Neurons Using Stereology

The length of NAergic axons were estimated using the stereological length estimation with spherical probes (Stereo Investigator; Micro Bright Field, Williston, VT, USA) [20,21]. Because of the regional variations in the densities of NAergic afferents, we focused our analysis on the selected subregions or ROIs (*S1BF* and dorsal hippocampus) for NAergic afferents. The total number of NAergic neurons in the LC was determine using the optical fractionator [20,21]. Every 4th section through the entire LC region was immunostained for TH and counterstained with CV. Total TH+ and TH− neuron numbers were estimated using the optical fractionator probe using the counting frame of 40 × 30 μm a 1 μm guard, a 130 × 130 μm sampling grid, and a dissector height of 10 µm. For unbiased stereological analysis of neuronal size (area and volume), we used the nucleator probe of the Stereo Investigator [20,21].

### 2.6. Synthesis of ψ-GSH

ψ-GSH (Figure S1) was synthesized in our laboratory using a previously described synthetic method [16]. Authentication of the compound was through NMR analysis and purity (>97% pure) of ψ-GSH was confirmed by reverse phase HPLC analysis.

### 2.7. Aβ_1–42_ ELISA Assay

The concentrations of Aβ_1–42_ in brain tissues were measured with a sandwich ELISA assay according to the manufacturer’s instructions (Thermo-Fisher, Waltham, MA, USA). Briefly, 50 μL of brain homogenate (PBS and guanidine fractions) and 50 µL of Hu Aβ_42_ Detection Antibody solution were added to each well, and the plate was then incubated for 3 h at ambient temperature. This was followed by individual 30 min incubation steps at room temperature with anti-rabbit IgG and a chromogenic substrate solution (100 μL/well) after brief washing steps. After quenching the reaction, the absorbance was measured at 450 nm using a Spectra Max M5 microplate reader. A standard curve of Aβ_1–42_ was generated to determine the concentration of Aβ_1–42_ in each sample and the data normalized to the weight of the tissue analyzed.

### 2.8. Quantitation of GSH in Mouse Brain

To measure GSH levels in mouse brain, we used a glutathione assay kit (Cayman Chemical, #703002, Ann Arbor, MI, USA). The protocol described in the kit was followed. Briefly, following a deproteinization step with metaphosphoric acid, the brain homogenates were neutralized to pH 6–7 with 4 M triethanolamine solution and diluted with 50 mM MES buffer (pH = 6.0, containing 1 mM EDTA) prior to their utilization in the assay. The kit is based on an enzymatic recycling method using GSH reductase, which allows reaction of the GSH sulfhydryl group with DTNB (5,5′-dithiobis-2-nitrobenzoic acid), producing a yellow-colored 5-thio-2-nitrobenzoic acid (TNB). For GSH disulfide measurement, deproteinated samples were incubated with 1M 2-vinylpyridine solution for 1 h at ambient temperature prior to the conduction of DTNB reactions.

### 2.9. Endogenous Brain Glo-1 Activities

Glo-1 activity was measured as described previously [16], with minor modifications. In brief, brain homogenates prepared in DPBS with proteinase inhibitors were used as Glo-1 and GSH source. Freshly distilled MG solution (2 mM) was incubated with the homogenate in 50 mmol/L sodium phosphate buffer (pH 6.6) for 10 min at 37 °C. Glo-1 activity was determined by measuring the formation of S-D-lactoylglutathione at 240 nm at 37 °C. The protein content was measured using the BCA assay (Thermo Scientific) and the enzyme activities were normalized to protein concentrations. To overcome the effect of reduced GSH levels affecting Glo-1 activity, the reaction mixtures were supplemented with excess GSH (2 mM), followed by measurement of S-D-lactoylglutathione formation at 240 nm.

### 2.10. Analysis of Methyglyoxal by LC-MS/MS

Methyglyoxal was determined using 1,2-diaminobenzene (DB) as a derivatizing agent as described previously [16,24], with the following modifications. Briefly, 20 µL of ice-cold TCA-saline, 40 µL of water, and 10 µL of 400 nM 2,3-hexanedione (Internal Standard) were added to 40 μL of mouse brain homogenates. The samples were centrifuged (10,000× *g* for 10 min at 4 °C) and supernatant was analyzed by LC-MS/MS. The LC-MS/MS conditions were as follows: Agilent 1260 HPLC (high–performance liquid chromatography) device coupled with an AB Sciex QTRAP 5500 mass spectrometer, Phenomenex Kinetex C18 column (50 × 2.1 mm, 2.6 µm), solvents-0.1% formic acid in water (mobile phase A), and 0.1% formic acid in acetonitrile (mobile phase B), flow rate of 0.5 mL/min. A positive mode electrospray ionization source was used for analyte detection. The following mass transitions were followed using multiple reaction monitoring (MRM): *m*/*z* 145.1→77.0 and 145.1→118 for derivatized methylglyoxal; *m*/*z* 187.1→158.1 for derivatized internal standard.

### 2.11. Quantitation of D-Lactate

Measurement of D-lactate levels was performed using D-lactate assay kit (Cayman Chemical, #700520) per manufacturer’s instructions. Briefly, the concentration of D-lactate in supernatants was determined by this assay in which, D-lactate dehydrogenase catalyzes oxidation of D-lactate to pyruvate, along with concomitant reduction of NAD^+^ to NADH [25]. The liberated NADH reacts with the fluorescent substrate, which is followed by excitation at 535 nm and emission at 590 nm. The results of this analysis were confirmed using an independent LC-MS/MS analysis described in the MG method with the following modifications. Deproteinized supernatants in 3% sulfosalicylic acid were used for this analysis. The chromatography conditions were as follows: Thermo Aquasil C18 column (150 × 2.1 mm, 3 µm, sovent-0.1% formic acid in water (mobile phase A) and 0.1% formic acid in acetonitrile (mobile phase B), flow rate of 0.4 mL/min. D-Lactate levels were determined using an electrospray ionization source operated in the negative mode using the following transition: *m*/*z* 89.1→43.0.

### 2.12. Western Blot Analysis

Mouse brain homogenates were prepared in RIPA buffer, complete with a protease inhibitor cocktail tablet [26]. The samples were denatured in Laemmli buffer (62.5 mM Tris–HCl, pH 6.8, 2% SDS, 10% glycerol, 5% 2-mercaptoethanol, 0.002% bromophenol blue). A fixed amount of protein was separated via SDS-PAGE and transferred to a polyvinylidene difluoride (PVDF) membrane. The membrane was probed for Glo-1 expression (anti-Glo-1 antibody, Abcam, Cambridge, UK) and AGEs (anti-AGE antibody, Millipore-Sigma, Burlington, MA, USA). The immunoreactivity we detected using horseradish peroxidase–linked antibody (Cell Signaling) and Clarity™ Western ECL substrate (Bio-Rad, Hercules, CA, USA) using the ChemiDoc™ MP Imaging System (Bio-Rad). Equal protein loading was confirmed by probing the membrane with anti-α-tubulin antibody.

### 2.13. ROS Assay

The ROS assay to assess oxidative stress was conducted as described previously [17]. Oxidation of 2′,7′-dichlorodihydrofluorescein diacetate (DCFH-DA) to fluorescent 2′,7′-dichlorofluorescein (DCF) in presence of ROS is measured in this assay. Supernatants of brain homogenates in DPBS were treated with DCFH-DA to a final concentration of 200 μM, and the mixtures were incubated at 37 °C in the dark for 60 min. Generation of the fluorescent DCF was assessed using a SpectraMax M5 microplate reader with excitation at 488 nm and emission at 525 nm. These values were normalized to total protein concentration in these samples.

### 2.14. Analysis of Lipid Peroxidation and Protein Carbonyl

Brain lipid peroxides was determined by colorimetric assay for malondialdehyde (MDA) (TBARS assay) [17] using the TBARS Assay Kit (Cayman Chemical Co., #10009055).

The protein carbonyl levels in mouse brain homogenates were determined using a protein carbonyl colorimetric assay kit (Cayman Chemical Co.) [17] following the manufacturers protocol. Following DNPH derivatization, absorbance of the supernatant corresponding to the amount of protein-hydrazone produced was measured at 370 nm using a Spectra Max M5. The absorbance was corrected by subtracting the absorbance of the samples without DNPH. The values then were normalized to protein concentration in these samples.

### 2.15. AGEs ELISA Assay

Mouse brain samples were prepared in DPBS with protease inhibitors as described before. The supernatants were assayed for AGE levels using a mouse AGE ELISA Kit (LifeSpan BioSciences, Seattle, WA, USA) [17]. Briefly, 100 μL of brain homogenate was incubated at 37 °C (90 min) in a 96-well plate. This was followed by incubations with 100 µL of biotin antibody and 100 µL of HRP-streptavidin with intermediate brief washing steps. Reaction with a chromogenic substrate solution (90 μL/well) was then followed by measuring absorbance at 450 nm using a Spectra Max M5 microplate reader. The levels of AGEs in each sample were calculated using a standard curve and normalized to the protein content of samples.

## 3. Results

### 3.1. Glo-1 Activity Is Reduced in APP/PS1 Mice and ψ-GSH Treatment Restores Glo-1 Activity

Previously, we showed that the indices of oxidative stress are increased in brains of young (6-mo old) female APP/PS1 mice and that ψ-GSH treatment was able to reverse increased oxidative stress [17]. However, it is currently unknown if Glo-1 activity is altered in transgenic mouse models of AD and whether ψ-GSH treatment can reverse the Glo-1 deficits as well as oxidative stress in aged APP/PS1 mouse model of AD.

**Figure 1:**
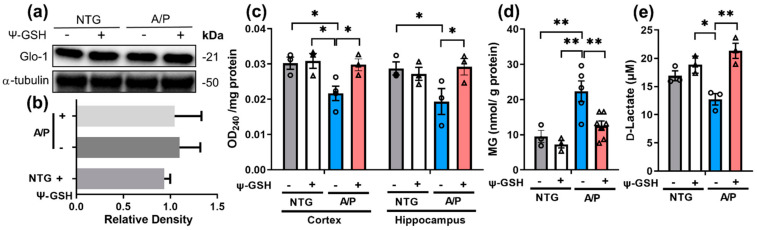
Restoration of Glo-1 enzyme activity after ψ-GSH treatment in symptomatic 14-mo-old APP/PS1 mice. Western blot analysis of the prefrontal cortex brain tissue of NTG and APP/PS1 (A/P) mice treated with saline (-) or ψ-GSH (+) for: (**a**) expression of Glo-1 protein and (**b**) quantification of Western blot in (**a**). (**c**) Endogenous Glo-1 activity was reduced in the cortex and the hippocampus of the vehicle-treated APP/PS1 mice compared to NTG controls. ψ-GSH treatment resuscitated Glo-1 function. (**d**) Increased MG levels in APP/PS1 mice and reduction in MG levels in ψ-GSH-treated APP/PS1 mice. (**e**) Levels of the product of the Glo-1 enzymatic reaction, d-lactate, were increased in the APP/PS1- ψ-GSH group compared to the saline controls, confirming in vivo enhancement of the Glo-1 pathway by ψ-GSH. Data are presented as the mean ± S.E.M. NTG, Saline- and ψ-GSH-treated APP/PS1 groups were compared with a one-way ANOVA with Tukey’s post-hoc test for statistical analysis. * *p* < 0.05, ** *p* < 0.01.

We first examined the Glo-1 protein levels in 14-mo-old APP/PS1 mice, which is expected to exhibit significant AD-like pathology. Immunoblot analysis showed that Glo-1 protein levels were not altered in APP/PS1 mice compared to the littermate NTG mice (Figure 1a,b). Further, ψ-GSH treatment did not affect the Glo-1 expression in brain. However, analysis of Glo-1 function showed that the endogenous Glo-1 activity was significantly reduced in both cortex and hippocampus of APP/PS1 mice (Figure 1c) and ψ-GSH treatment was able to reverse the loss of Glo-1 activity. To further determine changes specific to altered Glo activity in APP/PS1 mice, we measured the levels of MG in the brain. Our results showed that brain MG levels were significantly elevated in the 14-mo-old APP/PS1 mice compared to littermate nTg mice (Figure 1d). Moreover, ψ-GSH treatment was able to completely normalize the brain MG levels in APP/PS1 mice (Figure 1d). Consistent with the levels of MG in brain, analysis of Glo-1 activity in isolated brain lysates showed that the endogenous activity of Glo-1 and D-lactate generation, an enzymatic byproduct of Glo-1 reaction, was decreased in APP/PS1 mice compared to NTG mice (Figure 1e). Again, ψ-GSH treatment was able to reverse the Glo-1 deficit in APP/PS1 mice. Analysis of NTG mice shows that ψ-GSH treatment did not alter any of the parameters. These results show, for the first time, that a Tg mouse model of AD can recapitulate the loss of Glo-1 activity seen in human AD cases.

### 3.2. ψ-GSH Treatment in Aged APP/PS1 Mice Reverses Oxidative Stress

**Figure 2:**
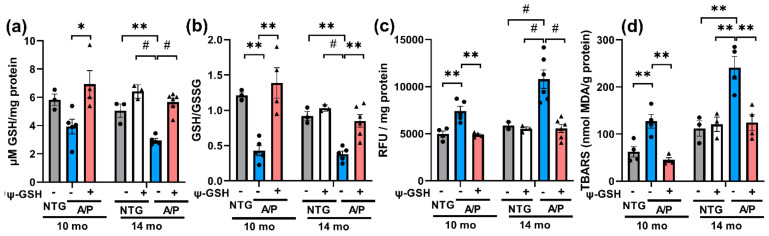
ψ-GSH treatment reverses oxidative stress in the APP/PS1 model. (**a**) ψ-GSH treatment restored the levels of reduced GSH to NTG controls. (**b**) Attenuation of oxidative stress was evident from enhanced redox potential in mice after ψ-GSH treatment in both symptomatic cohorts. (**c**) Total oxidative stress as measured by DCFH-DA assay was also mitigated by ψ-GSH in these mice irrespective of age. (**d**) TBARS results are shown as the mean ± S.E.M. Statistical significance was assessed by a one-way ANOVA with Tukey’s post-hoc test. * *p* < 0.05, ** *p* < 0.01, # *p* < 0.001.

We hypothesize that in APP/PS1 mice, as seen in human early AD cases [11], Glo-1 activity is reduced despite normal expression of the enzyme because of the reduced levels of cofactor GSH required for Glo-1 function. As a test of this hypothesis, we examined if GSH levels were indeed reduced in APP/PS1 mice. Our results showed that total GSH levels were reduced in the brain of APP/PS1 mice, particularly in the 14-mo-old mice (Figure 2a). Moreover, the GSH/GSSH ratio in 10 and 14-mo-old APP/PS1 mice were significantly lower than the nTg controls (Figure 2b), indicating reduced antioxidant capacity and increased oxidative stress in the APP/PS1 brain.

Reduction in brain GSH levels and GSH/GSSH ratio in APP/PS1 mice confirm the presence of ongoing oxidative stress with the progression of AD-like pathology. More importantly, we show that ψ-GSH can reverse GSH deficits. To provide additional evidence to support the view that ψ-GSH can reverse ongoing oxidative stress in aged APP/PS1 model, we also examined brain for other indices of oxidative stress, including ROS levels via DCFH-DA assay, lipid peroxidation using TBARS assay and protein oxidation by protein carbonyl levels (Figure 2c,d). Consistent with reduced GSH levels, levels of ROS and lipid peroxidation were significantly increased in the APP/PS1 mice. Further, ψ-GSH treatment completely reversed brain ROS and TBARS levels in APP/PS1 mice to the control levels (Figure 2c,d). In addition, we also examined the levels of AGE protein adducts resulting from increased MG and oxidative stress by immunoblot analysis for AGE. Our results show, for the first time, that AGE protein adducts are increased in 14-mo-old APP/PS1 mice and that this increase is normalized by ψ-GSH treatment (Figure S2). Similar to the results of AGE analysis, increased levels of protein carbonyls observed in 14-mo-old APP/PS1 mice brains were reduced compared to those in age-matched control mice after ψ-GSH treatment (Figure S2).

### 3.3. ψ-GSH Treatment Attenuates Aβ Burden and Reduces Neuroinflammatory Markers in Symptomatic APP/PS1 Mice

**Figure 3:**
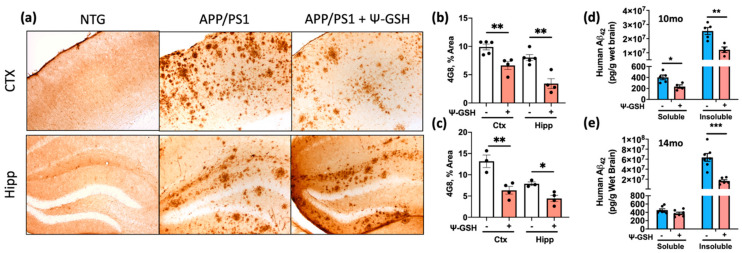
ψ-GSH treatment reduces Aβ burden in symptomatic 10-mo-old APP/PS1 mice. (**a**) Representative images of Aβ plaques visualized using 4G8 antibody in S1BF cortex and dentate gyrus of 14-mo NTG and APP/PS1 mice. Scale bar, 100 μm; (**b**) levels of Aβ plaques are significantly reduced in S1BF and hippocampus in ψ-GSH-treated 10-mo APP/PS1 mice (*n* = 5 APP/PS1 + Saline; *n* = 4 APP/PS1 + ψ-GSH); (**c**) levels of Aβ plaques are significantly reduced in S1BF and hippocampus in ψ-GSH-treated 14-mo APP/PS1 mice (*n* = 3 APP/PS1 + Saline; *n* = 4 APP/PS1 + ψ-GSH); (**d**,**e**) quantitation of the effect of ψ-GSH treatment on amyloid load in APP/PS1 mice using ELISA assay. Levels of insoluble Aβ_1–42_ levels in the brain homogenate of mice treated with ψ-GSH were significantly reduced in both 10-mo (**d**) and 14-mo (**e**) cohorts. For comparisons between saline and ψ-GSH-treated APP/PS1 groups (**b**,**c**), an unpaired Student’s *t*-test was performed for statistical analysis. * *p* < 0.05, ** *p* < 0.01, *** *p* < 0.005.

In young (6-mo old) APP/PS1 mice, ψ-GSH treatment reduced brain Aβ levels and delayed onset of Aβ deposition [17]. However, it is unknown if ψ-GSH treatment can impact Aβ pathology if the ψ-GSH treatment is initiated after the AD-pathology is well established. Because the APP/PS1 mice develop Aβ deposits by 6 mo of age, analysis of Aβ deposits using 4G8 staining of brain sections from 10- and 14-mo-old APP/PS1 mice showed severe Aβ pathology in both cortex and hippocampus (Figure 3, Figures S3 and S4). Significantly, age matched APP/PS1 mice treated with ψ-GSH for 2 mo exhibited obvious reduction in overall 4G8 immunoreactivity. Quantitative analysis of the S1BF region of the cortex and the hippocampus for the fraction of total area covered by Aβ-deposits confirmed that Ψ-GSH-treatment of aged APP/PS1 mice lead to a significant reduction in Aβ plaques, compared to their saline-treated APP/PS1 littermates (Figure 3a–c and Figure S3). A continuous intraperitoneal ψ-GSH treatment of 14 months-old APP/PS1 mice for 2 months also reduced the burden of insoluble Aβ_42_ species, marked by 12F4 antibody, compared to the saline-treated symptomatic APP/PS1 mice (Figure S4). As an additional confirmation that ψ-GSH reduces Aβ accumulation in aged APP/PS1 mice, we analyzed soluble and insoluble Aβ_42_ levels using a commercial ELISA assay. Our results showed that insoluble Aβ_42_ levels increased with age in APP/PS1 mice. With ψ-GSH treatment, insoluble Aβ_42_ levels were decreased in both 10-mo and 14-mo-old APP/PS1 mice (Figure 3d,e)

Glycation-induced oxidative stress is known to increase neuroinflammation and promotes accelerated Aβ aggregation and toxicity [5]. However, it is unknown if reduced glycation, as seen with ψ-GSH treatment, can also reduce neuroinflammation associated with Aβ pathology. Thus, we examined the expression of glial fibrillary acidic protein (GFAP) and the ionized calcium-binding adapter molecule 1 (Iba1) as markers of glial activation and inflammation in the brain of ψ-GSH- and saline-treated APP/PS1 mice as well as nTg littermates. We first examined if ψ-GSH treatment impacts glial reactivity in absence of any AD pathology in 14-mo-old NTG mice (Figure S5). Consistent with the biochemical data that showed no effects of ψ-GSH treatment in NTG mice (Figure 1 and Figure 2), our data show that the level of GFAP and Iba-1 immunoreactivity was not different in NTG mice as a function of ψ-GSH treatment (Figure S5).

**Figure 4:**
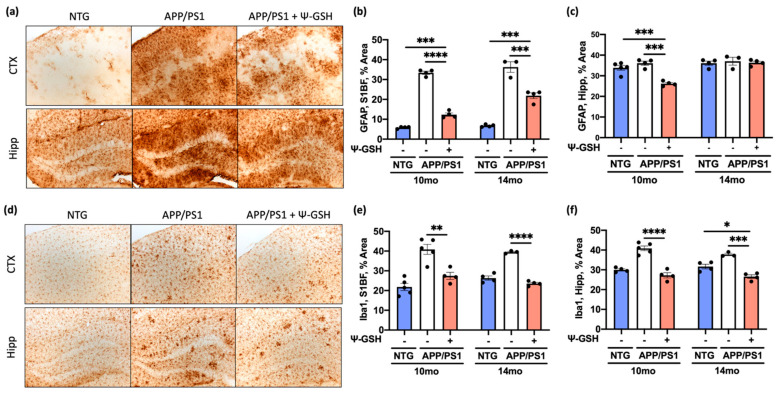
ψ-GSH treatment reduces reactive astrogliosis and microglial reactivity in symptomatic 10- and 14-mo APP/PS1 mice. (**a**) Representative images of reactive astrocytes visualized by GFAP antibody in dentate gyrus of 14-mo NTG and APP/PS1 mice. Scale bar, 100 μm. (**b**) Quantification of GFAP staining in S1BF of the 10-, 14-mo cohorts. (**c**) Quantification of GFAP staining in the hippocampus of the 10-, 14-mo cohorts. (**d**) Representative images of microglia visualized using Iba1 antibody in S1BF cortex of 14-mo NTG and APP/PS1 mice. Scale bar, 100 μm. (**e**) Quantification of Iba1 staining in S1BF of the 10-, 14-mo cohorts. (**f**) Quantification of Iba1 staining in the hippocampus of the 10-, 14-mo cohorts. Data are presented as mean ± S.E.M. For comparisons between NTG, saline-, and ψ-GSH-treated APP/PS1 groups, a one-way ANOVA with Tukey’s post-hoc test was used for data analysis. * *p* < 0.05, ** *p* < 0.01, *** *p* < 0.005, **** *p* < 0.001.

In APP/PS1 mice, Aβ deposits in brains of APP/PS1 mice were associated with significant increases in GFAP staining in the cortex of both 10-mo and 14-mo-old APP/PS1 mice (Figure 4a–c). Significantly, ψ-GSH-treated APP/PS1 brains exhibited significant reductions in the cortical GFAP staining at both 10 and 14 mo of age (Figure 4b). However, because the basal GFAP staining is very high in the hippocampus, we could not document increased GFAP in the hippocampus of 14-mo-old APP/PS1 mice (Figure 4c). Analysis of brain sections immunostained for microglia, using anti-Iba1 antibody, revealed that overall abundance of microglia was increased with Aβ pathology in both cortex and hippocampus of APP/PS1 mice (Figure 4d). Consistent with the effects of ψ-GSH treatment on Aβ deposition and astrocyte activation, ψ-GSH treatment of APP/PS1 mice lead to significant reductions in microglial staining in the brains of both the 10 and 14 months-old APP/PS1 mice (Figure 4e,f and Figure S6a,b).

**Figure 5:**
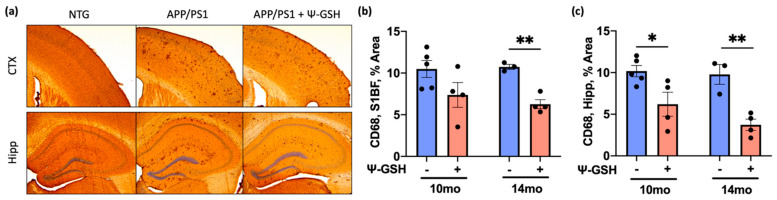
ψ-GSH treatment effectively reduces CD68-positive activated microgliosis in symptomatic 14-mo-old APP/PS1 mice. (**a**) Representative images of CD68 antibody positive microglia in the S1BF region of the cortex and the dorsal hippocampus of 14 months-old saline-treated NTG and APP/PS1 mice treated with either saline or ψ-GSH. (**b**) Quantification of CD68-positive immunostained area coverage of the S1BF region of the cortex in 10- and 14-mo-old APP/PS1 mice either treated with saline or ψ-GSH. (**c**) Quantification of CD68-positive immunostained area coverage of the hippocampus in 10- and 14-mo APP/PS1 mice either treated with saline or ψ-GSH. For comparisons between saline and ψ-GSH-treated APP/PS1 groups (**b**,**c**), an unpaired Student’s *t*-test was performed for statistical analysis. * *p* < 0.05, ** *p* < 0.01.

To selectively analyze activated microglia, we stained the tissue sections for CD68, which is highly expressed in activated microglia. In the 10- and 14-mo-old APP/PS1 mice, the CD68 staining is prominent while almost no CD68 staining is seen in nTg littermates (Figure 5). Consistent with the Iba1 staining, ψ-GSH treatment significantly reduced inflammatory and activated microglia from APP/PS1 mice (Figure 5a–c). The Iba1 and CD68 immunostaining show that ψ-GSH treatment attenuates microglial activation in the brain of APP/PS1 mice. Collectively, these data suggest that ψ-GSH treatment induces protective mechanisms including anti-inflammatory and antioxidant responses, which then contribute to the reduction of pathological Aβ load, even in a late-stage, symptomatic mouse model of AD.

### 3.4. ψ-GSH Treatment Protects against Progressive Noradrenergic Neurodegeneration

**Figure 6:**
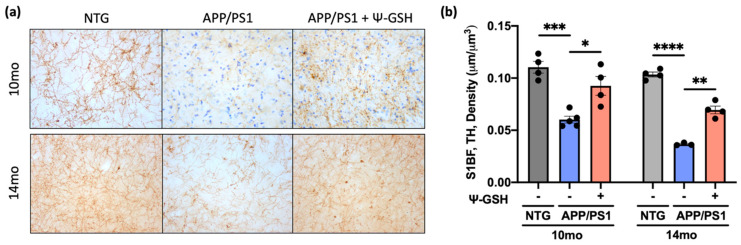
ψ-GSH treatment after the onset of AD-like neuropathology halts the progressive degeneration of the NAergic cortical afferent axons in symptomatic 10- and 14-mo APP/PS1 mice. (**a**) Representative images of TH+ axonal projections in S1BF of both the 10- and the 14-mo cohorts. Scale bar, 20 μm. (**b**) Quantification of TH+ axonal density in 10- and 14-mo NTG and APP/PS1 mice treated with either saline or ψ-GSH. Density estimation (μm/μm^3^) was determined using the Spherical probe on Stereo Investigator Software. Data are presented as mean ± S.E.M. A one-way ANOVA with Tukey’s post-hoc test was used for statistical comparisons (**b**). * *p* < 0.05, ** *p* < 0.01, *** *p* < 0.005, **** *p* < 0.001.

Patients with both idiopathic and familial forms of AD exhibit early neurodegeneration in the brainstem, particularly the cluster of nuclei in the locus coeruleus (LC), even before the onset of cognitive impairment or Aβ plaque development [27,28]. The severity of neuronal loss in the LC often correlates with the duration of AD symptoms and the selective and early vulnerability of the neurons in the LC may contribute to deficits in memory and attention. Previously, we demonstrated that the degeneration of the LC neurons seen in human AD is recapitulated in APP/PS1 mice [20]. Thus, we examined whether ψ-GSH treatment can attenuate progressive degeneration of NAergic neurotransmitter system in APP/PS1 mice. Consistent with the lack of Aβ pathology in NTG mice, comparison of ψ-GSH-treated NTG mice with saline-treated NTG mice for NAergic afferents and axons did not reveal any differences (Figure S5). However, analysis of the cortical NAergic afferents in APP/PS1 mice shows significant loss of TH+ fibers in 10-mo-old APP/PS1 mice that progresses to greater loss in 14-mo-old APP/PS1 mice (Figure 6). Significantly, ψ-GSH treatment rescued the cortical TH+ axon loss even when the treatment started after the onset of neurodegeneration (Figure 6). In particular, the fact that TH+ axon density in ψ-GSH-treated APP/PS1 mice at 14 mo of age is comparable to that seen in 10-mo-old APP/PS1 mice indicate that ψ-GSH was able to completely stop the progression of further neurodegeneration.

**Figure 7:**
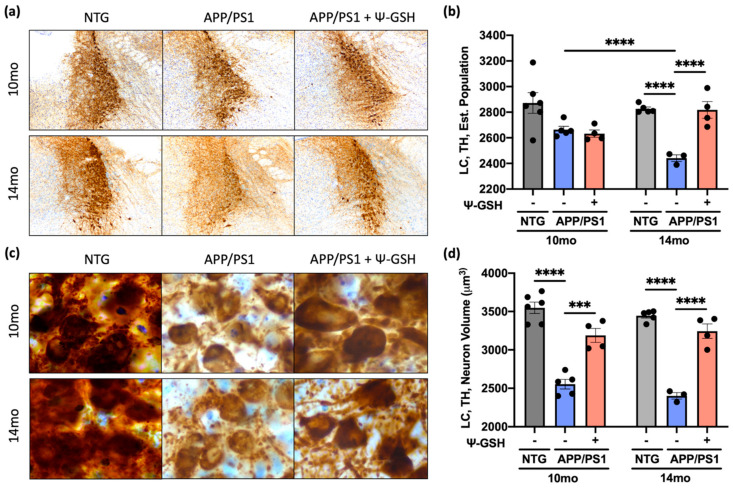
ψ-GSH treatment after the onset of AD-like neuropathology halts the progressive degeneration of the NAergic neurons of the LC in symptomatic 10 and 14-mo APP/PS1 mice. (**a**) Representative images of TH+ neurons of the LC in both the 10 and 14-mo cohorts. Scale bar, 100 μm. (**b**) Quantification of TH+ neurons in the LC in 10- and 14-mo NTG and APP/PS1 mice treated with either saline or ψ-GSH. Estimated population was determined by the Optical Fractionator probe on Stereo Investigator software. (**c**) Higher magnification images of the TH+ neurons in the LC. Scale bar, 50 μm. (**d**) Quantification of the TH+ neuronal volume in 10- and 14-mo NTG and APP/PS1 mice treated with either saline or ψ-GSH. Data are presented as mean ± S.E.M. A one-way ANOVA with Tukey’s post-hoc test was used for statistical comparisons (**b**,**d**). *** *p* < 0.005, **** *p* < 0.001.

We previously showed that Aβ deposition leads to initial loss of NA axons followed by atrophy of cell bodies LC and finally the loss of TH+ neurons in the LC [20].Thus, we examined whether ψ-GSH treatment also reversed the progressive neuronal atrophy and loss in APP/PS1 animals. Consistent with previous study showing that the loss of neurons occurs after 12 mo of age in APP/PS1 mice, there was no significant loss of LC neurons at 10-mo-old APP/PS1 mice compared to nTg controls. However, the loss of cortical afferents at 10-mo-old APP/PS1 mice (Figure 6) is reflected by the decrease in neuronal volumes of TH+ cells in the LC (Figure 6a,b and Figure 7c,d). While ψ-GSH treatment had no impact on the overall TH+ neuronal counts in the 10 mo-old APP/PS1 mice (Figure 7a,b), the neuronal volumes were significantly higher with ψ-GSH treatment (Figure 7c,d). In the 14-mo-old APP/PS1 mice, there was a significant loss of TH+ neurons and reduced neuronal volumes in LC (Figure 7). Significantly, in the 14-mo-old APP/PS1 mice treated with ψ-GSH from 12 mo of age, progressive loss of TH+ neurons in LC was completely abated (Figure 7a,b). Surprisingly, while ψ-GSH treatment between 12 and 14 mo still showed reduced cortical TH+ afferents, comparable to mice at 10 mo of age, ψ-GSH treatment was able to completely reverse the decrease in neuronal volume seen in APP/PS1 mice at both 10 and 14 mo of age. These data demonstrate the capacity of continuous ψ-GSH treatment, delivered intraperitoneally, to protect against NAergic neurotransmitter network even in the late stage of AD pathology and symptoms. This may, in turn, be associated with the reduction of Aβ burden and glial activation seen in ψ-GSH-treated APP/PS1 mice.

## 4. Discussion

We show that AD-like neuropathology in the APP/PS1 mouse model of AD is associated with multiple indices of oxidative stress, including decreased GSH/GSSH levels, decreased Glo-1 function, increased MG, and increased carbonyls. Treatment of these animals that have already developed progressive pathology with a GGT-resistant GSH analog, Ψ-GSH, was able to completely reverse all indicators of oxidative stress in APP/PS1 mice. Neuropathological analysis shows that Ψ-GSH treatment also attenuated progression of Aβ deposition and neuroinflammation. Finally, we show that ψ-GSH treatment attenuates progressive degeneration of NAergic neurons in the LC. Collectively, our results show that the APP/PS1 mouse model recapitulates features seen in human AD, including accumulation of oxidative stress, MG/AGE accumulation, neuroinflammation, and progressive degeneration of LC neurons. Further, Ψ-GSH treatment, even when applied after AD-like pathology is established, can reverse oxidative stress and attenuate progressive AD pathology, including inflammation and neurodegeneration.

Oxidative stress is established as a key pathogenic component in AD, contributing to both disease onset and progression [29]. While the source of oxidative stress in human AD is complex and multifactorial, results from Tg mouse model of amyloid pathology show that amyloid pathology is sufficient to initiate the pathological process leading to increased oxidative stress in the brain [30]. Moreover, robust oxidative stress in the APP/PS1 mouse model used here occurs at early stages of AD pathology [17], prior to the presence of robust neuroinflammation and the onset of neurodegeneration. Thus, the APP/PS1 model used here will be valuable for defining how Aβ abnormalities cause increased oxidative stress and how oxidative stress in AD causes progressive neurodegeneration.

One of the most abundant anti-oxidants in brain is the GSH. In AD brains and in mouse models of AD, numerous studies have established significant reduction in GSH and/or GSH/GSSH ratio with AD pathology, indicating that antioxidant capacity of the brain is overwhelmed with AD pathology [31]. Imbalance between accumulated reactive oxygen species and innate antioxidant response leads to increased free radicals resulting in feed forward cycles of oxidative stress, cellular dysfunction, and inflammation. In addition, while glucose is the major energy source for brain, glucose metabolism generates toxic reactive MG which can lead to covalent modifications of proteins in forms of AGEs [5,6]. Increased oxidative stress promotes formation of MG and AGEs, which also contribute to increase of Aβ plaques, tau tangles, and neuroinflammation [5,6]. Significantly, in addition to acting as the major antioxidant in brain, GSH is also directly involved in preventing accumulation of MG by acting as an essential cofactor for Glo-1 enzymatic reaction. In our APP/PS1 model, while Glo-1 expression is not altered, loss of brain GSH is associated with impaired Glo-1 function. This scenario recapitulates the status of Glo-1 in early AD brain where Glo-1 activity and MG levels are increased despite of the increase in Glo-1 expression [11].

To date, utilization of general antioxidants such as polyphenols, vitamins E and C, to mitigate oxidative stress observed in AD has met with limited clinical success [32,33,34]. Such treatments have offered promising results in preclinical AD models, however clinical efficacy evaluations are still inconclusive. A more target-oriented antioxidant strategy is expected to overcome the limitations of general antioxidants and presents a two-pronged approach to tackle biochemical changes observed in AD. We put forth a novel strategy by supplementing brain’s own detoxification machinery by directly and more efficiently increasing brain GSH levels using a novel GSH analog, Ψ-GSH, which normalizes increased oxidative stress and restores Glo-1 activity. While N-acetylcysteine (NAC), a precursor for GSH synthesis, has been shown to increase brain GSH levels, supplementation of NAC or l-cysteine levels may be ineffective in aging brain due to glutamylcysteine ligase (GCL) deficiency in the aging brain [35]. Preclinical studies in rodent models and human “Nutraceutical” trials show that NAC could be beneficial for AD, however, evidence supporting the beneficial effect of NAC alone in human AD cases is relatively weak as NAC supplementation alone leads to limited benefits in human studies [36]. Administration of γ-glutamylcysteine [37], in the brain penetrant diethyl ester [38], may address GCL deficiency but not the efficacy limiting GGT-mediated catabolism. While direct chemical scavengers of oxidized sugars could be helpful [39], the scavenger amounts needed exceed rational or safe dosing. Our strategy was to supplement intracellular GSH levels by synthesis of GGT-stable GSH analog, ψ-GSH, that possesses improved pharmacokinetic profile and is able to substitute for GSH in Glo-1 enzymatic reaction [16]. Our previous study demonstrated the utility of this strategy and efficacy of ψ-GSH as a preventative treatment for AD in APP/PS1 mice [17]. This report shows the therapeutic potential of this approach to alter the ongoing AD pathology and related biochemical changes, including progressive neurodegeneration. Given the similarities of the parameters examined here to those seen in early AD, we are optimistic that the findings from this study will be translated to human AD. It is important to note that the lack of neurodegenerative studies in preclinical studies using AD models likely contributed to general failure of preclinical studies to translate to successful therapies for AD patients. Thus, our results showing that Ψ-GSH can attenuate ongoing neurodegeneration in AD model provides increased confidence that the results here will be more relevant to clinical efficacy.

## 5. Conclusions

In summary, reduced GSH, dysfunctional Glo-1 activity, and oxidative stress seen in human early AD cases are recapitulated in the APP/PS1 mouse model of AD. The fact that supplementation with Ψ-GSH attenuates all of the pathological parameters in aged APP/PS1 models show that reduced brain GSH level is a key driver of amyloid-dependent pathological changes, including progressive degeneration of LC neurons. Our results also show for the first time, ψ-GSH efficiently engaged with the target Glo-1 enzyme and counteracted glycation-induced oxidative and inflammatory pathology even in animals with ongoing pathology and neurodegeneration. Thus, Ψ-GSH represents a viable strategy for development of future AD therapeutics and supports further investigations for advancement of ψ-GSH to tackle this devastating disease. Moreover, because oxidative stress and GSH depletion are common features of many neurodegenerative diseases, it will be important to determine if ψ-GSH treatment can attenuate disease in models of tauopathy and α-synucleinopathy.

## Data Availability

All raw data or original images of gels/histology will be made available upon request by More and Lee.

## Supplementary Materials

The following are available online at https://www.mdpi.com/article/10.3390/antiox10111796/s1. Supplementary File 1 (Supplementary Figures S1–S6.).
