# Supplementary material for: γ-Glutamyl-Transpeptidase-Resistant Glutathione Analog Attenuates Progression of Alzheimer’s Disease-like Pathology and Neurodegeneration in a Mouse Model
Source: Antioxidants (Basel). 2021 Nov 10;10(11):1796. doi: 10.3390/antiox10111796 (PMC8614797; doi:10.3390/antiox10111796)
Supplement: Supplementary file 1 [file antioxidants-10-01796-s001.zip › antioxidants-1426616-supplementary.pdf]

Supplementary File 1

Supplementary Figures S1-S6

For

γ-Glutamyl-Transpeptidase Resistant Glutathione Analog Attenuates Progression of Alzheimer's disease-like Pathology and Neurodegeneration in a Mouse Model.

Ye In Christopher Kwon, Wei Xie, Haizhou Zhu, Jiashu Xie , Keaton Shinn, Nicholas Juckel, Robert Vince, Swati S. More , Michael K. Lee

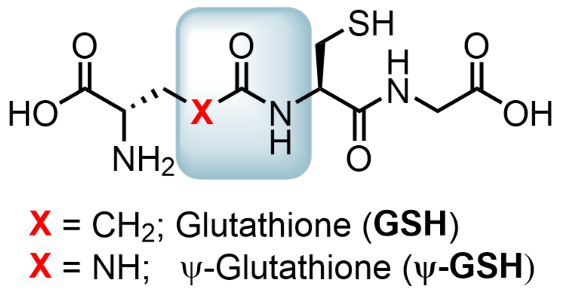

Figure S1. Chemical structure showing the difference between GSH and ψ-GSH. Natural GSH is degraded by ubiquitously expressed γ-glutamyl transpeptidase (GGT), limiting its bioavailability. By replacing the labile γ-glutamyl-cysteinyl amide bond with a urea linkage (highlighted in the supporting information), we have obtained a GGT-resistant GSH analogue, ψ-GSH, which is recognized by all GSH-dependent enzymes.

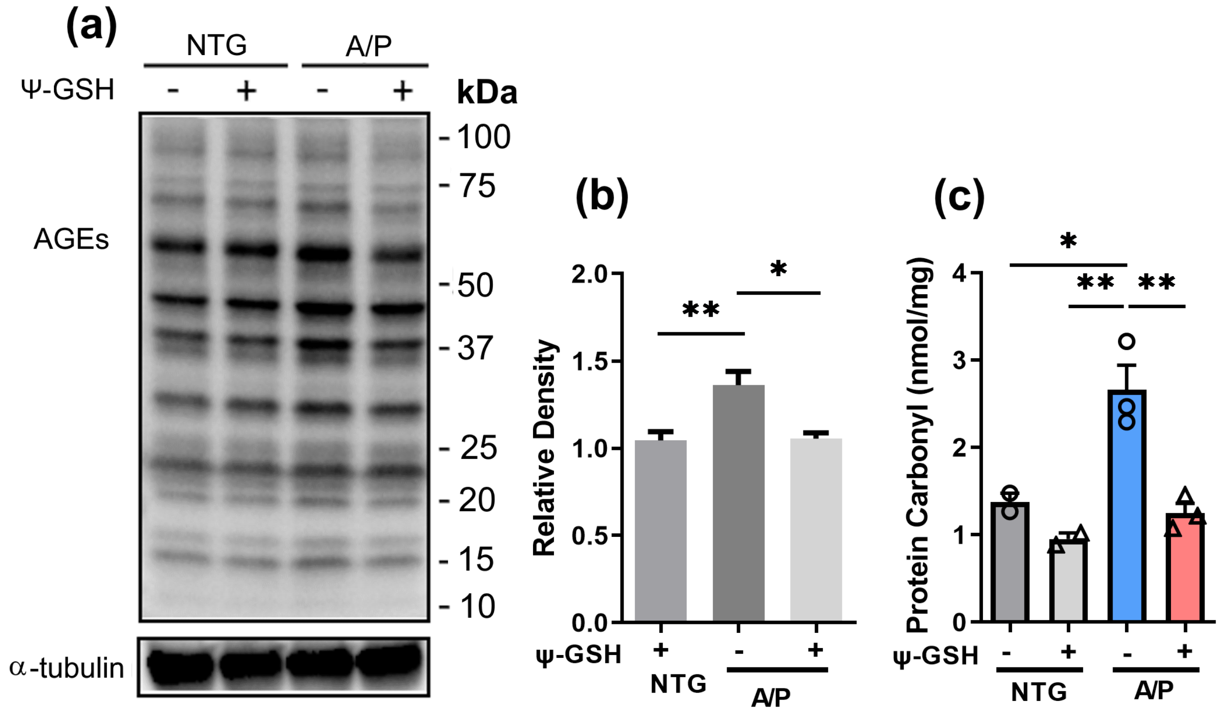

Figure S2. (a) Western blot analysis of the prefrontal cortex of NTG and APP/PS1 mice treated with saline and ψ-GSH for levels of AGEs (b). (b) Quantification of Western blot in (a) showed higher accumulation of AGEs in APP/PS1 mouse brains, which were significantly reduced after ψ-GSH treatment. For comparisons between NTG, saline-, and ψ-GSH-treated APP/PS1 groups, a one-way ANOVA with Tukey's post-hoc test was used. *p<0.05, **p<0.01.

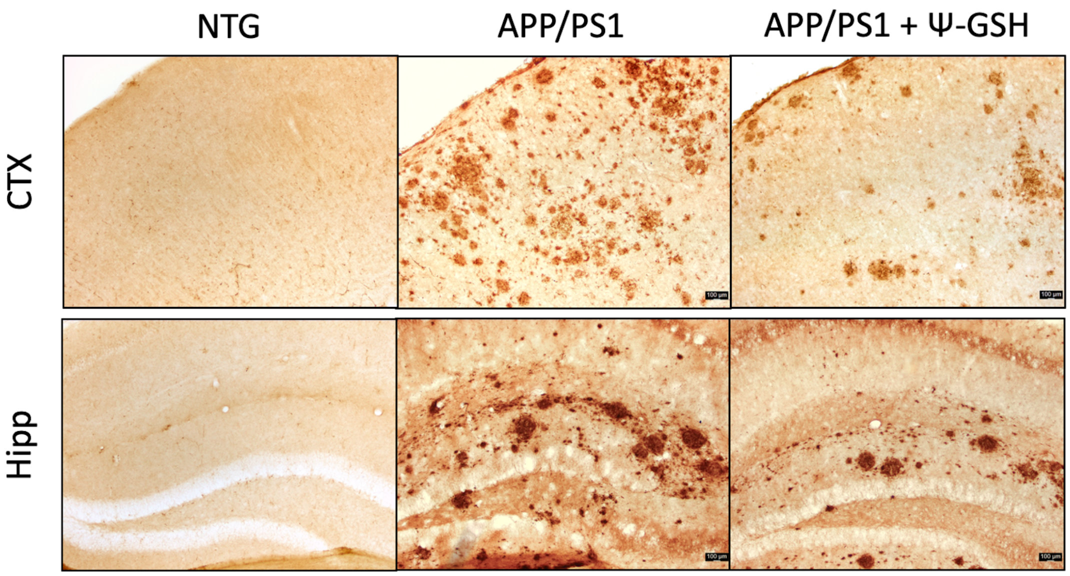

Figure S3. ψ-GSH treatment reduces Aβ burden in symptomatic 10mo old APP/PS1 mice. (a) Representative images of Aβ plaques visualized using 4G8 antibody in S1BF cortex and dentate gyrus of NTG and APP/PS1 mice. Scale bar, 100μm.

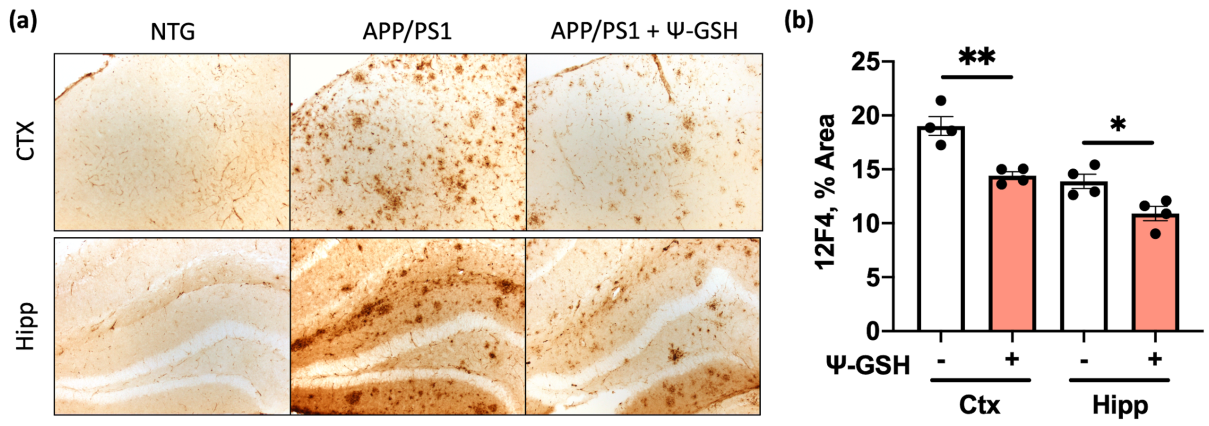

Figure S4. ψ-GSH treatment reduces $A\beta_{x\text{-}42}$ species in symptomatic 14mo APP/PS1 mice. (a) Representative images of $A\beta_{x\text{-}42}$ visualized using 12F4 antibody in S1BF cortex and dentate gyrus of NTG and APP/PS1 mice. Scale bar, 100μm; (b) Quantification of $A\beta_{x\text{-}42}$ staining in S1BF and hippocampus. For comparisons between saline and ψ-GSH-treated APP/PS1 groups (b), an unpaired Student's *t*-test was performed for statistical analysis. **p*<0.05, ***p*<0.01.

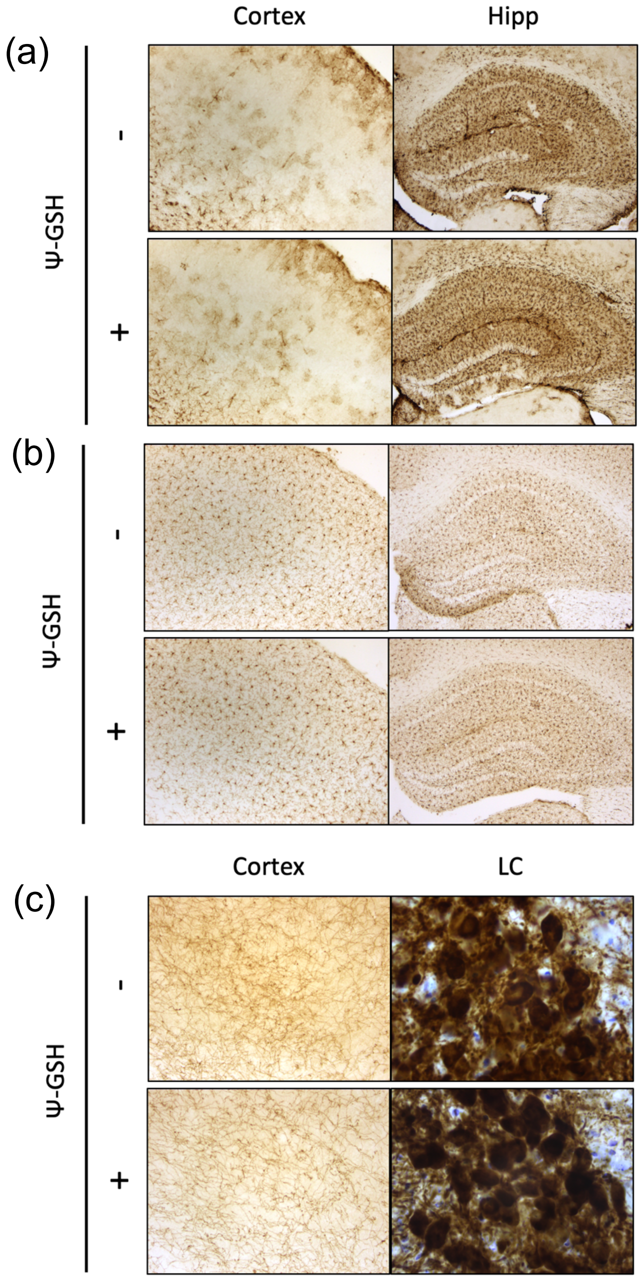

Figure S5. ψ-GSH treatment has no impact on glia response and NAergic integrity in absence of Aβ pathology in NTG mice. Representative images of brain sections from NTG mice, treated with saline or Ψ-GSH, immune-stained for GFAP (a), Iba1(b), and TH(c). Quantitative analysis of S1BF show similar abundance of GFAP (Sal:ψ-GSH, 5.7±0.9: 5.25±0.2% area); Iba1 (Sal:ψ-GSH, 16.8±1.9: 15.5±2.1 % area); and TH fibers (Sal:ψ-GSH, $8.8 \times 10^{-3} \pm 0.8 \times 10^{-3}$: $8.1 \times 10^{-3} \pm 0.9 \times 10^{-3}$ μm/μm^3). Analysis of LC shows similar size (Sal:ψ-GSH, 3645± 332:3665±301 μm^3) and number (Sal:ψ-GSH, 2826± 55: 2872±101 TH+ neurons) of TH+ neurons in both groups. In all cases, the differences in the means were not significant (student *t*-test). Comparable results were found with the analysis of hippocampus.

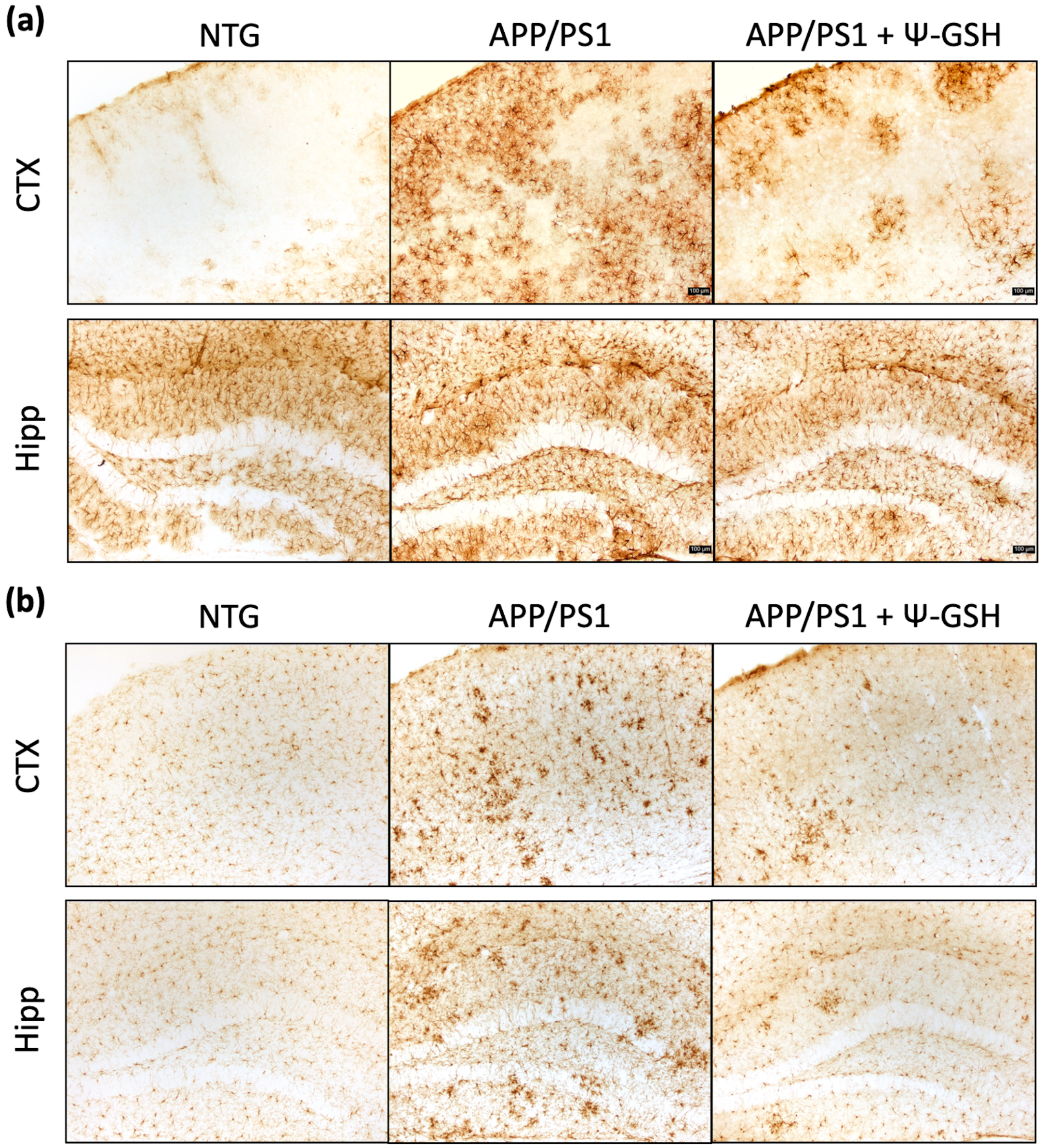

Figure S6. ψ-GSH treatment reduces reactive astrogliosis and microglial reactivity in symptomatic 10mo old APP/PS1 mice. (a) Representative images of reactive astrocytes visualized by GFAP antibody in dentate gyrus of 10mo NTG and APP/PS1 mice. Scale bar, 100μm; (b) Representative images of microglia visualized by Iba1 antibody in dentate gyrus of 14mo NTG and APP/PS1 mice. Scale bar, 100μm.
